# Clinical Utility of Pepsin and Bile Acid in Tracheal Secretions for Accurate Diagnosis of Aspiration in ICU Patients

**DOI:** 10.3390/jcm12175466

**Published:** 2023-08-23

**Authors:** Dirk Bandorski, Khodr Tello, Harun Erdal, Janine Sommerlad, Jochen Wilhelm, Istvan Vadasz, Matthias Hecker, Dieter Walmrath, Werner Seeger, Ekaterina Krauss, Stefan Kuhnert

**Affiliations:** 1 Faculty of Medicine, Semmelweis University Campus Hamburg, Lohmühlenstraße 5/Haus P, 20099 Hamburg, Germany; 2 Intensive Care Medicine and Internal Diagnostics, Neurological Clinic Bad Salzhausen, 63667 Nidda, Germany; 3 Department of Internal Medicine, Justus Liebig University, Universities of Giessen and Marburg Lung Center (UGMLC), 35392 Giessen, Germany; 4 German Center for Lung Research (DZL), 35392 Giessen, Germany; 5 The Cardio-Pulmonary Institute (CPI), 35392 Giessen, Germany; 6 Institute for Lung Health (ILH), 35392 Giessen, Germany; 7 Department of Lung Development and Remodeling, Max Planck Institute for Heart and Lung Research, 61231 Bad Nauheim, Germany

**Keywords:** intensive care, aspiration, pneumonia

## Abstract

Background: Aspiration of stomach content or saliva in critical conditions—e.g., shock, intoxication, or resuscitation—can lead to acute lung injury. While various biomarkers in bronchoalveolar lavage fluids have been studied for diagnosing aspiration, none have been conclusively established as early indicators of lung damage. This study aims to evaluate the diagnostic value of pepsin, bile acid, and other biomarkers for detecting aspiration in an intensive care unit (ICU). Materials and methods: In this study, 50 ICU patients were enrolled and underwent intubation before admission. The evaluation of aspiration was based on clinical suspicion or documented instances of observed events. Tracheal secretion (TS) samples were collected within 6 h after intubation using sterile suction catheters. Additional parameters, including IL-6, pepsin, and bile acid, were determined for analysis. Pepsin levels were measured with an ELISA kit, while bile acid, uric acid, glucose, IL-6, and pH value in the tracheal secretion were analyzed using standardized lab methods. Results: The 50 patients admitted to the ICU with various diagnoses. The median survival time for the entire cohort was 52 days, and there was no significant difference in survival between patients with aspiration pneumonia (AP) and those with other diagnoses (*p* = 0.69). Among the AP group, the average survival time was 50.51 days (±8.1 SD; 95% CI 34.63–66.39), while patients with other diagnoses had a mean survival time of 32.86 days (±5.1 SD; 95% CI 22.9–42.81); the survival group comparison did not yield statistically significant results. The presence of pepsin or bile acid in TS patients did not significantly impact survival or the diagnosis of aspiration. The *p*-values for the correlations between pepsin and bile acid with the aspiration diagnosis were *p* = 0.53 and *p* > 0.99, respectively; thus, pepsin and bile acid measurements did not significantly affect survival outcomes or enhance the accuracy of diagnosing aspiration pneumonia. Conclusions: The early and accurate diagnosis of aspiration is crucial for optimal patient care. However, based on this study, pepsin concentration alone may not reliably indicate aspiration, and bile acid levels also show limited association with the diagnosis. Further validation studies are needed to assess the clinical usefulness and reliability of gastric biomarkers in diagnosing aspiration-related conditions. Such future studies would provide valuable insights for improving aspiration diagnosis and enhancing patient care.

## 1. Introduction

The aspiration of stomach content or saliva is a concerning complication in acute situations, such as shock, intoxication, or resuscitation. Aspiration refers to the entry of pharyngeal secretions, food particles, or gastric secretions into the larynx and trachea, leading to their descent into the lungs. This process triggers an inflammatory response known as aspiration pneumonia (AP) [1]. As a result of acute lung injury, which initiates a series of inflammatory reactions involving the recruitment of inflammatory cells and the release of inflammatory mediators, AP can rapidly progress to respiratory failure [2,3]. Consequently, patients diagnosed with AP experience higher levels of morbidity and higher mortality rates than those patients diagnosed with community-acquired pneumonia [4].

Since its initial documentation in the medical literature during the 19th century, the syndrome of pulmonary aspiration of gastric contents has continued to attract substantial attention in various clinical domains. The occurrence of pulmonary aspiration poses a significant health risk for hospitalized patients, with reported mortality rates ranging from 10% to 71% [5,6,7].

In 1999, a definition of AP was provided by Professor Sekizawa and the committee members of the Japanese Respiratory Society (JRS), as follows: “AP is diagnosed following confirmation of inflammatory findings in the lungs and overt aspiration (apparent aspiration), a condition in which aspiration is strongly suspected, or the existence of dysphagia is confirmed” [8]. In the official clinical practice guidelines of the American Thoracic Society and Infectious Diseases Society of America, no definition separates patients with AP from those with typical pneumonia [9].

Teramoto et al. indicated that AP comprises two pathological conditions: airspace infiltration with bacterial pathogens and dysphagia-associated mis-swallowing [10]. Furthermore, the condition may occur due to an impaired swallowing function, which should be classified as (a) apparent aspiration that causes choking while swallowing during meals or (b) silent aspiration of unwitnessed throat and periodontal secretions [11].

Clinically important episodes of aspiration have been reported to occur in approximate 25% of intubated patients [12]. Due to these factors, and in the context the acute events, early administration of antibiotic therapy is typically practiced in intensive care units (ICUs). Although certain studies have made progress in identifying risk factors for AP, healthcare providers still face challenges when it comes to the clinical presentation and diagnosis of this condition [13]. The exact prevalence of AP has never been accurately determined, as a substantial portion of aspiration episodes(up to 37%) are described as “silent”, “occult”, or unwitnessed [14].

Currently, in cases where clinical evidence of aspiration is not apparent, there is a lack of validated diagnostic methods to confirm its occurrence. Extensive research has been conducted on various biomarkers that are present in bronchoalveolar lavage fluids (BALF) for the diagnosis of aspiration. However, no biomarker has been definitively established to indicate early-stage lung damage caused by aspiration.

In particular, there is a lack of markers that can differentiate between AP of bacterial and chemical origin. For instance, the presence of bile acid and pepsin in the lungs is unexpected in normal conditions; therefore, no standard levels have yet been established for these biomarkers [15]. As far as our knowledge extends, despite the growing availability of gastric biomarkers for aspiration, none of these biomarkers has undergone profound clinical validation. 

A reliable biochemical marker for detecting pulmonary aspiration of gastric contents should typically not be present in significant quantities in the tracheobronchial tree or lungs, and its presence should be limited to gastric secretions. Pepsin, which is produced as an inactive pre-enzyme (called pepsinogen) by specialized cells in the gastric mucosa, fulfills these criteria. While minimal amounts of pepsin may be absorbed into the systemic circulation, its concentrations in the airway and lungs should be minimal [16].

Badellino et al. developed a rabbit model of induced pulmonary aspiration, where peptic activity was detected in bronchoalveolar lavage fluid (BALF) up to 60 min after aspiration of gastric contents; no peptic activity was found in control animals subjected to aspiration of normal saline solution. Furthermore, peptic activity was detected in all eight animals 15 min after aspiration, but by 60 min only five of the eight animals demonstrated measurable peptic activity in tracheobronchial fluid. A time-dependent decrease in peptic activity/mL lavage fluid was noted in samples that were positive for peptic activity [17].

Therefore, there remains a need for further research and validation to determine the clinical efficacy of gastric biomarkers in diagnosing aspiration and guiding appropriate treatment decisions in clinical settings [18].

This prospective observational study, conducted at the Universities of Giessen and Marburg Lung Center (UGMLC) site in Giessen, aimed to investigate the frequency of AP in ICU patients who underwent on-site orotracheal intubation before being admitted. This study further aimed to compare the diagnostic value of pepsin in tracheal secretion (TS) with clinical suspicion or observed aspiration events.

Additionally, this research sought to assess the additional predictive value of pepsin in TS for AP and early ventilator-associated pneumonia, as well as to explore the potential impact of pepsin detection on in-hospital mortality. Moreover, this study aimed to evaluate pepsin and bile acids in TS as markers for the early detection of aspiration, comparing their performance with the current standards for observed events or clinical suspicion. Finally, the distribution of uric acid, IL-6, and glucose in tracheal secretions was analyzed as a potential biomarker for the early diagnosis of aspiration pneumonia.

## 2. Materials and Methods

This prospective observational study included a cohort of 50 ICU patients who met the following inclusion criteria: (1) the patients’ admissions were not related to surgical or gynecological reasons, (2) the patients’ ages ranged from 18 to 85 years, (3) there was a clinical indication for pre-clinical or emergency room intubation before ICU admission at Medical Clinic II of the UGMLC site in Giessen, (4) a blockable tracheal tube was used, and (5) TS samples, collected within 6 h after orotracheal intubation, were available.

Exclusion criteria were as follows: (1) an unstable circulatory situation, such as high-grade arrhythmias; (2) critical ventilatory settings with FiO_2_ > 80%, PEEP > 15 mmHg, and/or P_insp_ > 30 mmHg; pre-existing antibiotic therapy; (3) advanced lung diseases such as lung fibrosis with long-term oxygen therapy or chronic obstructive pulmonary disease (COPD, GOLD stage > III); (4) intubation due to known pneumonia or infection; and (5) patients with potential chronic aspiration, such as those with a recent stroke (<8 weeks) or neuromuscular diseases, pre-existing complete dependence on care, or pre-existing non-invasive ventilation. In addition, patients who had undergone lung transplantation or gastrectomy were excluded from the study.

Within 6 h of orotracheal intubation, the following diagnostic procedures were conducted for the study patients admitted to the ICU: clinical microbiological routine examination of TS; documentation of study parameters, including biometric data, co-morbidities, and medication; and suspected or observed aspiration by the emergency ambulance team. Additionally, ventilation parameters (FiO_2_, tidal volume, minute ventilation, PEEP) and clinical routine parameters (blood values, microbiology, X-ray findings) were recorded. If a patient required bronchoscopy with BALF, the results were considered in the evaluation, and measurements of pepsin, bile acid, IL-6, pH value, uric acid, glucose, and triglycerides were performed, adhering to the study protocol.

TS samples were collected in the ICU by the study investigator within 6 h after orotracheal intubation, using sterile suction catheters. The sample-processing procedure involved using approximately 2.5 mL of a TS sample for routine microbiological diagnostic, followed by withdrawal of 2.5 mL into a heparin monovette for IL-6 analysis and using a serum monovette for bile acid determination. Approximately 2 mL of the remaining TS sample was transferred to an Eppendorf reaction vessel for pepsin measurement. The remaining sample was utilized to analyze pH values, triglycerides, urea, and glucose. Pepsin in the TS sample was detected using a commercially available ELISA test system. Condensed water collected from the ventilation system (breath condensate) was withdrawn into a 2 mL syringe (minimum 1 mL) to determine the pH value. If immediate processing of the samples was not possible, the entire sample tube was stored at −20 °C in the ICU and sent to the laboratory on the next working day.

The measurements and samples obtained were primarily intended for routine diagnostic and patient care purposes. In the ICU, TS is a standard procedure used to clear airway obstructions and collect samples for routine microbiological diagnosis. However, for the purpose of this study, additional parameters, such as IL-6, pepsin, and bile acid, were determined. It is worth noting that the insertion of a nasogastric tube is a common clinical practice for reflux control measures. In addition, no further blood samples were required for this study. Furthermore, the regular removal of condensed water from the ventilator’s hose system is a necessary procedure to ensure proper functioning. Moreover, no additional examinations, including X-rays or blood withdrawals, were carried out on the patients. All procedures were conducted under the supervision of ICU medical staff, eliminating the need for additional analgesic sedation.

The laboratory staff used a commercially available ELISA kit to detect human pepsin in blood and secretion samples. The pH value in the breath condensate was assessed by collecting condensed water from the ventilator’s hose system, preferably after at least 12 h of ventilation. For the determination, a minimum of 1 mL of condensed water was collected in a sealed 2 mL syringe and sent to the central laboratory, labeled as “breath condensate”, for pH analysis. The central laboratory staff employed standardized methods to determine bile acid, uric acid, glucose, IL-6, and the pH value in the TS samples.

During days 1 to 14 after admission, follow-up documentation of the clinical routine parameters (such as blood values, microbiology, and, if relevant, chest X-ray results) were documented daily, along with continuous monitoring of ventilation parameters (fraction of inspired oxygen, FiO_2_, tidal volume, minute ventilation, and PEEP). The presence of aspiration within four days after intubation was assessed through clinical observations, including the identification of newly occurring infiltrates in the chest X-ray.

The study adhered to the Helsinki Declaration, GCP Ordinances (GCP-V), and ICH-E6 guidelines for good clinical practice. Ethical approval was obtained from the ethics committee of Justus-Liebig University of Giessen, Germany (235/11). Each patient was assigned a unique identification number (a pseudonym) and provided informed consent to participate, with the freedom to withdraw at any time without providing reasons. Termination of participation, if applicable, was documented, including the time and the reasons. Relevant clinical findings, predominantly collected as routine data, were marked and stored, according to the study protocol.

### Statistical Methods

Statistical procedures were performed using SPSS 29 (SPSS, IBM Corp., Armonk, NY, USA) and R 3.4 (https://www.R-project.org, accessed on 2 May 2017). Descriptive statistics were used to present demographic values and the distribution of aspiration markers in TS, providing estimated mean values and standard deviations. The distribution of various aspiration markers was presented in tabular form, including the number of samples or observations for each marker, the minimum and maximum values recorded, and the mean values and standard deviations. Comparative analyses were performed using t-tests and chi-square tests. Survival analysis was conducted using Cox regression and presented as Kaplan–Meier curves.

## 3. Results

### 3.1. Demographics and Descriptive Characteristic of Study Cohort

This study analyzed a total of 50 patients admitted to the ICU at UGMLC site Giessen for various reasons, encompassing acute respiratory failure (*n* = 2), aspiration (*n* = 5), intoxication (including heroin) (*n* = 6), resuscitation (*n* = 35), myocardial infarction (*n* = 1), and acute pancreatitis (*n* = 1). The mean age of the patients at baseline was 61.76 ± 17.2 years. Of the total cohort, 32 (60%) were male. The descriptive statistics are presented in Table 1, summarizing and providing a structure and better readability for presenting the mean values and standard deviations of the parameters.

### 3.2. Survival in Dependency on Presence of Aspiration Pneumonia

**Figure 1:**
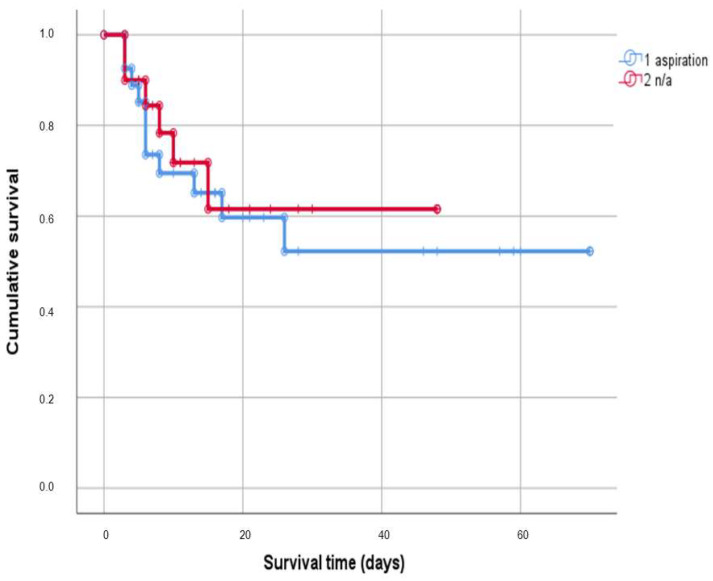
Survival in dependency of aspiration pneumonia. Abbreviations: n/a—not any.

Figure 1 presents the survival outcomes within a cohort of patients. The median survival time for the entire cohort of patients, including cases where the exact survival time was unknown (censored cases), was 52 days. The 95% confidence interval (CI) for the median survival ranged from 38.68 to 63.38.

Within the study cohort, 28 patients received a diagnosis of AP, defined as a newly occurring infiltrate in the chest X-ray, as well as increases in inflammation parameters and in ventilation invasiveness. The average survival time in this group was 50.51 days (±8.1 SD; 95% CI 34.63–66.39). The ICU patients with other diagnoses showed a mean survival time of 32.86 days (±5.1 SD; 95%CI 22.9–42.81). The group comparison showed a *p*-value of 0.69, indicating that there was no statistically significant difference in survival between the two groups. Still, this information is valuable for understanding the overall survival duration.

### 3.3. Effect on Survival from the Detection of Pepsin in the Tracheal Secretion

**Figure 2:**
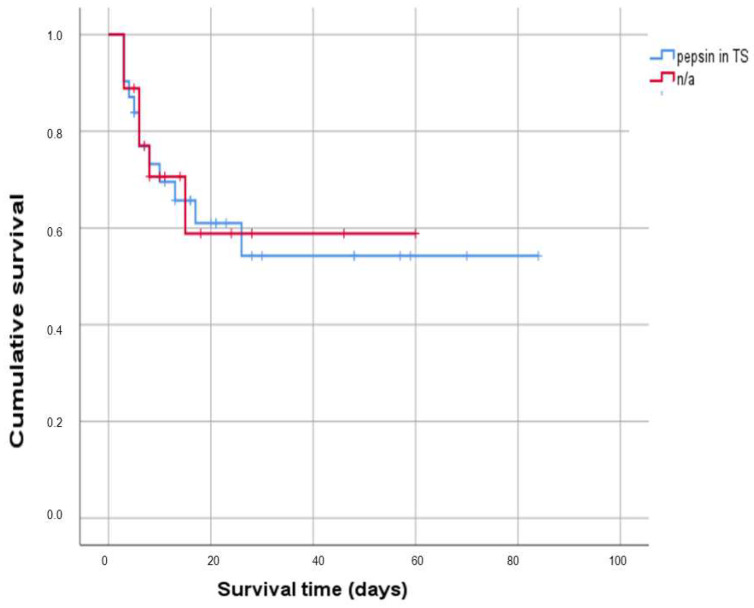
Survival in cases in dependency on the presence of pepsin in tracheal secretion. Abbreviations: n/a—not any.

The impact of pepsin detection in the TS on outcome was assessed using survival analysis. In the pepsin-negative group, the average survival time was 44.52 days (±8.9 SD; 95% CI 26.97–62.01). Conversely, ICU patients with positive pepsin in their tracheal secretion had a mean survival time of 52.26 days (±7.7 SD; 95% CI 37.22–67.3). However, the survival comparison between these two groups did not yield statistically significant results, as indicated by the *p*-value of 0.92. Figure 2 presents Kaplan–Meier curves.

### 3.4. The Value of the Pepsin and Bile Acid in TS as Markers for an Early Detection of Aspiration, Compared with Observed Events or Clinical Suspicion

The analysis examined the relationship between pepsin and bile acid levels and the clinical diagnosis of AP, thus testing their additional predictive value as biomarkers and comparing their performance with the current standard, i.e., observed aspiration events or clinical suspicion. We examined the correlation between the diagnosis of aspiration (“yes”/“no”) and the levels of pepsin and bile acid in TS.

There was no pronounced association observed between pepsin levels and the diagnosis of aspiration. The dots representing pepsin values were evenly distributed across the entire measuring range, regardless of the diagnosis. This suggests that the concentration of pepsin alone may not be a reliable indicator of the presence or absence of aspiration. 

**Figure 3:**
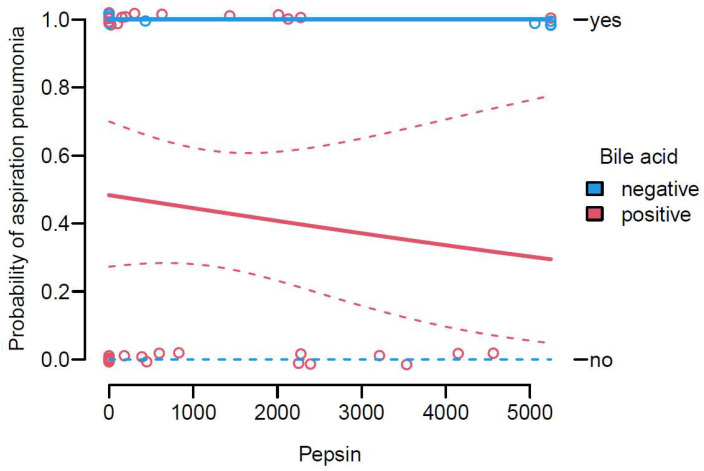
Correlation between pepsin and bile acid levels in TS, compared to the clinical and radiological AP diagnosis.

Furthermore, the analysis showed that the correlation between bile acid values and the diagnosis of aspiration was not prominently evident. The red-color-coded dots representing bile acid values were spread across the measuring range, indicating no clear association between bile acid levels and the diagnosis of aspiration. As presented in Figure 3, for patients with negative bile acid, the confidence band extended over the entire range, indicating maximum uncertainty in the prediction. Despite this, all patients with negative bile acid results had a positive clinical diagnosis of AP. In patients with negative bile acid, there were cases with both positive and negative AP diagnoses, and this pattern was consistent across different pepsin values. The number of cases with positive and negative diagnoses was roughly the same, indicating that the probability of a positive AP diagnosis for patients with positive bile acid was approximately 50%. Furthermore, the logistic model also resulted in the following *p*-values for the relationships between pepsin and bile acid with the diagnosis: pepsin, *p* = 0.53; bile acid, *p* > 0.99. Therefore, knowledge of pepsin (*p* = 0.53) did not significantly improve the estimation of the probability of diagnosis, when compared to knowledge of bile acid (*p* = 0.52).

In an example scenario, the relationship between pepsin values and bile acid values and the probability of a positive diagnosis was examined. For a patient with a pepsin value of 2000 and negative bile acid value, there was a 95% CI, indicating that the probability of a positive diagnosis ranged from 0% to 100%. However, a positive diagnosis was more likely than a negative diagnosis in this case, and the same result would be obtained for patients with pepsin values of 0 or 10. On the other hand, if a patient has a pepsin value of 2000 and a positive bile acid value, the 95% confidence interval suggested that the probability of a positive diagnosis was between 20% and 60%. Again, similar results would be obtained for patients with pepsin values of 0 or 10. Overall, the knowledge of pepsin or bile acid did not effectively help to estimate the probability of a positive diagnosis. Furthermore, neither the predictive value of pepsin in TS for early ventilator-associated pneumonia nor the analysis of uric acid, IL-6, and glucose distribution in TS as potential biomarkers for early AP diagnosis yielded statistically significant results.

## 4. Discussion

This study investigated the role of pepsin and bile acid in TS as potential markers for early detection of AP. The results showed no significant association between pepsin levels and the diagnosis of aspiration.

Pepsin values were evenly distributed across the entire range, suggesting that pepsin concentration alone may not accurately indicate the presence or absence of aspiration. Similarly, the correlation between bile acid values and the diagnosis of aspiration was not prominent. The statistical model analysis also showed that pepsin did not significantly improve the estimation of the probability of AP diagnosis, compared to bile acid. Overall, the knowledge of pepsin or bile acid alone did not effectively help in estimating the probability of a positive AP diagnosis, and relying solely on pepsin or bile acid may not be sufficient for accurately predicting the likelihood of a positive diagnosis.

To assess the impact of pepsin detection in TS on patient outcomes, a survival analysis was conducted. The average survival time for patients in the pepsin-negative group was found to be 44.52 days, while the average survival time was slightly higher, at 52.26 days, for patients in the pepsin-positive group. However, it is important to note that the comparison of survival between these two groups did not yield statistically significant results. The *p*-value of 0.92 suggested that the difference in survival between the pepsin-negative and pepsin-positive groups could be attributed to chance, rather than to a direct association with pepsin levels.

This study implemented further specific criteria in AP recognition, including the evaluation of chest X-rays for new infiltrates and the assessment of inflammation and ventilation parameters, with the aims of ensuring a consistent and accurate identification of AP within the study cohort and contributing to the reliability and accuracy of the diagnosis process. Ufberg et al. reported an incidence of aspiration of 38.5% in trauma patients intubated outside the clinical setting, while pepsin could be detected in 50% of the patients examined; however, a small number of aspiration events were investigated (*n* = 20) [19]. In another publication by the same group, the presence of pepsin in the TS of patients intubated outside the hospital was explored [20]. D’Ovidio et al. demonstrated that elevated levels of bile acid in the BALF were associated with increased levels of neutrophils and IL-8, as well as with reduced surfactant phospholipids; furthermore, those patients exhibited a higher prevalence of positive BALF cultures for fungi and bacteria [21].

Hashemi-Bajgani et al. conducted a study in which they detected bile acid and pepsin in the BALF of all patients, with mean concentrations of 29.84 ± 4.30 µmol/L and 113.17 ± 13.10 ng/mL [22]. In our study, we observed mean values for the digestive enzymes that were different from those in the study by Hashemi-Bajgani et al. Specifically, for pepsin, the mean value was 1517.64 ng/mL with a standard deviation of 1826.95 ng/mL, while the mean concentration of bile acid was 1.64 µmol/L with a standard deviation of 1.60 µmol/L. We also measured the mean values of lipase (5.67 ng/mL) and amylase (5961.36 ng/mL), with their respective standard deviations and ranges.

These differences highlight the variations in enzyme concentrations between the two studies and may reflect differences in patient populations, sample collection methods, or other factors (e.g., age, underlying health conditions, or the presence of specific diseases that can influence enzyme levels). Alternatively, potential variations in sample collection methods might contribute to the differences observed. Factors such as the timing and the techniques of sample collection, storage conditions, and processing protocols can impact enzyme concentrations. Even slight variations in these procedures can lead to differences in measured values. Additionally, differences in laboratory methodologies, including the specific assays used to quantify enzyme concentrations, could contribute to the variations. Each study may have employed different techniques or equipment, leading to potential discrepancies in the reported values. Furthermore, variations in the analytical instruments and reagents used in the assays could affect the accuracy and precision of the measurements, leading to differences in the reported concentrations.

Overall, the variations in enzyme concentrations between our study and the study by Hashemi-Bajgani et al. likely resulted from a combination of patient-related factors, differences in sample collection and processing methods, laboratory techniques, and inherent biological variability. Different laboratory methods may have varying sensitivities and detection limits, influencing the duration during which pepsin can be detected following aspiration events. Understanding these factors is crucial for interpreting and comparing the findings of different studies in the field.

As an example, the study by Ufberg et al. showed that the duration of the detectability of pepsin depends on the method used. Pepsin activity in vivo may decrease with time and the testing may, somehow, not remain sensitive for more than 30 to 60 min after the event of aspiration. The authors discussed a novel assay or technique that determined the presence of pepsin in respiratory samples [20]. The concentration profile of pepsin, following aspiration, is not well understood [17]. The study by Jaoude et al. investigated various biomarkers, including pepsin, to assess their utility in diagnosing aspiration-related conditions. Understanding the temporal changes in biomarker concentrations after aspiration is crucial for accurate diagnoses and for the management of aspiration syndromes [23].

Metheny et al. reported that BALF pepsin concentrations change over time following an aspiration event, and elevated levels may reflect less dilution at the time of sampling, high-volume and/or high-frequency aspiration events, or impaired clearance by the lung itself. Their results showed that 92.9% of pepsin-positive specimens were obtained from subjects in a flat position. Considering that pepsin in TS as an indicator of gastric content aspiration, it was concluded that aspiration occurred in five of the 30 study subjects [24]. In a rabbit model of induced pulmonary aspiration, Badellino et al. showed that peptic activity can be detected in the bronchoalveolar fluid for up to 60 min after aspiration of gastric contents [17]. This finding shed light on one possible reason why diagnostic tests involving biomarkers such as pepsin have shown poor sensitivity and specificity in some other studies [23,25].

In an analysis of 115 claims of malpractice of pulmonary aspiration, Warner et al. showed that death occurred in 57% of the cases and severe permanent injury occurred in another 14% of the cases. Clinical risk factors included emergency procedures, major trauma, gastrointestinal obstruction, acute intraabdominal processes, obesity with body mass indices greater than or equal to 35, recent food intake, recent opioid administration, gastroesophageal reflux disease, diabetes, previous gastric bypass or sleeve, history of neurologic disease (stroke, depressed consciousness, or a neurologic condition that may impair oropharyngeal coordination, such as Parkinson’s disease), and current pregnancy [26]. Moreover, clinical care issues included non-placement of a nasogastric tube before the aspiration event, poor clinical management of the aspiration event, and difficult intubation (i.e., more than three attempts) [26].

To effectively manage the risk of aspiration, a comprehensive approach encompasses refined assessments, innovative airway techniques, skilled personnel, risk stratification, collaboration, clear protocols, and vigilant monitoring. Enhancements in assessing both airway and gastric contents, refining airway management strategies, and ensuring the presence of skilled personnel to manage patients with increased aspiration risk are all avenues to improve the outcome of patients who are susceptible to aspiration events.

The economic implications of this study are important in clinical decision-making and healthcare resource allocation, which revolve mostly around cost reduction. If pepsin and bile acid measurements do not notably enhance the accuracy of diagnosing aspiration pneumonia, healthcare facilities can save costs by avoiding unnecessary laboratory tests, thereby reducing the potential for erroneous outcomes that might trigger unnecessary treatments. By underscoring that pepsin and bile acid measurements do not significantly boost diagnostic accuracy for aspiration pneumonia, this study provides insights to guide clinicians and healthcare administrators in making prudent choices about diagnostic testing. Consequently, this leads to cost savings, optimal use of resources, and improved patient care.

The different methodologies used in the above-mentioned studies likely capture different aspects of aspiration-related events and diagnostic approaches. The limited accuracy of these tests suggests their inability to reliably detect all cases of aspiration or to differentiate them from other respiratory conditions and highlights the need for the development of more reliable and effective methods. By addressing these limitations, future research can focus on improving the accuracy of diagnostic tests, ensuring that they can accurately detect aspiration and differentiate it from other respiratory conditions. This will lead to more precise and targeted administration of antibiotics, enhancing patient care and optimizing antibiotic stewardship.

## 5. Conclusions

The early and accurate diagnosis of aspiration is crucial for effective antibiotic treatment and optimal patient care. However, this study suggests that the concentration of pepsin alone may not reliably indicate aspiration, and the levels of bile acid also do not show a strong association with the diagnosis. It is evident that the current diagnostic tests, including those involving pepsin or bile acid biomarkers, have limitations in detecting all cases of aspiration and differentiating them from other respiratory conditions. Therefore, additional research is needed to develop more effective diagnostic methods and to address these limitations.

Further validation studies might provide valuable insights into the role of these biomarkers in improving the accuracy and efficiency of aspiration diagnosis, aiming to optimize diagnostic precision and, ultimately, to enhance patient care.

## Figures and Tables

**Table 1:** Distribution of aspiration markers in the TS in the study cohort.

	*n*	Mean Value	SD	Minimum	Maximum
Digestive Enzymes					
Pepsin	48	1517.64	1826.95	0	5246.1
Lipase	6	5.67	7.312	0	20
Bile acid	45	1.64	1.60	0.1	7.5
Amylase	14	5961.36	15,326.14	0	58,000
Lipids and metabolic markers					
Triglyceride	46	36.20	83.137	0	334
Glucose	46	8.15	14.272	0	73
Inflammatory markers					
IL6	50	940.27	2626.24	0	13,506
Other markers					
Protein	45	3.61	12.52	0	84
LDH	30	790.43	1846.99	0	8329
Cholesterin	3	1.67	2.082	0	4
Uric acid	47	6.77	10.92	0	55
pH measurements					
pH tracheal secretion	40	7.01	0.38	6	7.76
pH condensate		6.44	0.49		

Abbreviations: SD—standard deviation; *n*—number of samples or observations; pH—potential of hydrogen; IL-6—interleukin-6; LDH—lactate dehydrogenase.

## Data Availability

Data is available upon request.

## References

[1] Ficke B., Rajasurya V., Cascella M. (2020). StatPearls: Chronic Aspiration.

[2] Hunt E., Sullivan A., Galvin J., MacSharry J., Murphy D. (2018). Gastric Aspiration and Its Role in Airway Inflammation. Open Respir. Med. J..

[3] Dragan V., Wei Y., Elligsen M., Kiss A., Walker S.A.N., A Leis J. (2018). Prophylactic Antimicrobial Therapy for Acute Aspiration Pneumonitis. Clin. Infect. Dis..

[4] Neill S., Dean N. (2019). Aspiration pneumonia and pneumonitis. Curr. Opin. Infect. Dis..

[5] Hamelberg W., Bosomworth P.P. (1964). Aspiration pneumonitis: Experimental studies and clinical observations. Anesth. Analg..

[6] Lewis R.T., Burgess J.H., Hampson L.G. (1971). Cardiorespiratory Studies in Critical Illness. Changes in aspiration pneumonitis. Arch. Surg..

[7] Cameron J.L., Mitchell W.H., Zuidema G.D. (1973). Aspiration Pneumonia. Clinical outcome following documented aspiration. Arch. –Surg..

[8] Sekizawa K., Matsui T., Nakagawa T., Nakayama K., Sasaki H. (1998). ACE inhibitors and pneumonia. Lancet.

[9] Metlay J.P., Waterer G.W., Long A.C., Anzueto A., Brozek J., Crothers K., Cooley L.A., Dean N.C., Fine M.J., Flanders S.A. (2019). Diagnosis and treatment of adults with community-acquired pneumonia. An official clinical practice guideline of the american thoracic society and infectious diseases society of America. Am. J. Respir. Crit. Care Med..

[10] Teramoto S., Yoshida K., Hizawa N. (2015). Update on the pathogenesis and management of pneumonia in the elderly-roles of aspiration pneumonia. Respir. Investig..

[11] Teramoto S. (2021). The current definition, epidemiology, animal models and a novel therapeutic strategy for aspiration pneumonia. Respir. Investig..

[12] Kingston G.W., Phang P., Leathley M.J. (1991). Increased incidence of nosocomial pneumonia in mechanically ventilated patients with subclinical aspiration. Am. J. Surg..

[13] Rodriguez A.E., Restrepo M.I. (2019). New perspectives in aspiration community acquired Pneumonia. Expert Rev. Clin. Pharmacol..

[14] Haleem M.A. (1990). Aspiration pneumonia as a cause of death. Int. J. Clin. Pract..

[15] Blondeau K., Mertens V., Vanaudenaerde B.A., Verleden G.M., Van Raemdonck D.E., Sifrim D., Dupont L.J. (2008). Gastro-oesophageal reflux and gastric aspiration in lung transplant patients with or without chronic rejection. Eur. Respir. J..

[16] Samloff I.M. (1981). Pepsins, peptic activity, and peptic inhibitors. J. Clin. Gastroenterol..

[17] Badellino M.M.M., Buckman R.F.J., Malaspina P.J., Eynon C.A.M., O’Brien G.M., Kueppers F. (1996). Detection of pulmonary aspiration of gastric contents in an animal model by assay of peptic activity in bronchoalveolar fluid. Crit. Care Med..

[18] Lee A.S., Ryu J.H. (2018). Aspiration Pneumonia and Related Syndromes. Mayo Clin. Proc..

[19] Ufberg J.W., Bushra J.S., Karras D.J., Satz W.A., Kueppers F. (2005). Aspiration of gastric contents: Association with prehospital intubation. Am. J. Emerg. Med..

[20] Ufberg J.W., Bushra J.S., Patel D., Wong E., Karras D.J., Kueppers F. (2004). A new pepsin assay to detect pulmonary aspiration of gastric contents among newly intubated patients. Am. J. Emerg. Med..

[21] D’ovidio F., Mura M., Tsang M., Waddell T.K., Hutcheon M.A., Singer L.G., Hadjiliadis D., Chaparro C., Gutierrez C., Pierre A. (2005). Bile acid aspiration and the development of bronchiolitis obliterans after lung transplantation. J. Thorac. Cardiovasc. Surg..

[22] Hashemi-Bajgani S.-M., Abbasi F., Shafahi A., Yazdani R., Samareh Fekri M. (2019). Association of Bile Acid and Pepsin Micro-aspiration with Chronic Obstructive Pulmonary Disease Exacerbation. Tanaffos.

[23] Jaoude P.A., Knight P.R., Ohtake P., El-Solh A.A. (2010). Biomarkers in the diagnosis of aspiration syndromes. Expert Rev. Mol. Diagn..

[24] Metheny N.A., Chang Y.-H., Ye J.S., Edwards S.J., Defer J., Dahms T.E., Stewart B.J., Stone K.S., Clouse R.E. (2002). Pepsin as a Marker for Pulmonary Aspiration. Am. J. Crit. Care.

[25] Dhanapal S.G., Vijay J., Amirtharaj J., Ganesan P. (2021). Aspiration during Rapid Sequence Induction: Prevalence and Risk Factors. Indian J. Crit. Care Med..

[26] Warner M.A., Meyerhoff K.L., Warner M.E., Posner K.L., Stephens L., Domino K.B. (2021). Pulmonary Aspiration of Gastric Contents: A Closed Claims Analysis. Anesthesiology.

